# Clinical Outcomes of Lateral Lumbar Interbody Fusion with Percutaneous Pedicle Screw for Dialysis-Related Spondyloarthropathy

**DOI:** 10.3390/jcm13041089

**Published:** 2024-02-14

**Authors:** Shigeyuki Kitanaka, Ryota Takatori, Hitoshi Tonomura, Yuichi Shimizu, Masateru Nagae, Atsushi Makinodan, Kenji Takahashi

**Affiliations:** 1 Department of Orthopedic Surgery, Nishijin Hospital, Kyoto 602-8319, Japan; makinodan@nishijin.net; 2 Department of Orthopaedics, Graduate School of Medical Science, Kyoto Prefectural University of Medicine, Kyoto 602-0841, Japan; r-taka@koto.kpu-m.ac.jp (R.T.); tono@koto.kpu-m.ac.jp (H.T.); y-shimi@koto.kpu-m.ac.jp (Y.S.); sho-gun@koto.kpu-m.ac.jp (M.N.); orthoped@koto.kpu-m.ac.jp (K.T.)

**Keywords:** hemodialysis, lateral lumbar interbody fusion, percutaneous pedicle screw, bone fragility

## Abstract

**Background**: The usefulness and problems with lateral lumbar interbody fusion (LLIF) with a percutaneous pedicle screw (PPS) for dialysis-related spondyloarthropathy are not clear. Therefore, we investigated the usefulness and problems with LLIF with PPS in dialysis-related spondyloarthropathy. **Methods**: In total, 77 patients who underwent LLIF with PPS were divided into two groups: the dialysis-related spondyloarthropathy group (“Group D”) consisted of 15 patients (10 males and 5 females) with a mean age of 70.4 years and a mean duration of hemodialysis of 10.8 years; and the lumbar degenerative disease group (“Group L”) included 62 patients (31 males and 31 females) with a mean age of 71.0 years. The mean follow-up period was 4 years in Group D and 3 years 9 months in Group L. We compared surgical invasiveness (operative time, blood loss), perioperative complications, clinical outcomes (Improvement ratio of the JOA score), bone fusion rate, reoperation, sagittal alignment, and coronal imbalance between the two groups. **Results**: There were no significant differences in operative time, blood loss, or the improvement ratio of the JOA score, but dialysis-related spondyloarthropathy was observed in one patient with superficial infection, three patients with endplate failure, and one patient with restenosis due to cage subsidence. **Conclusions**: We consider LLIF with PPS for dialysis-related spondyloarthropathy to be an effective treatment option because its surgical invasiveness and clinical outcomes were comparable to those for cases of lumbar degenerative disease. However, as endplate failure due to bone fragility and a reduced bone fusion rate were observed in dialysis spondylolisthesis cases, we advise a careful selection of indications for indirect decompression as well as the application of suitable pre- and postoperative adjuvant therapies.

## 1. Introduction

With the development of medical technology for hemodialysis, the life expectancy of dialysis patients has been extended and the number of long-term dialysis patients has been increasing. Kuntz et al. [1] first reported destructive spondyloarthropathy (DSA) as a spinal disease occurring in long-term hemodialysis patients. Since then, various other findings in addition to DSA have been observed in the spines of hemodialysis patients. In DSA, amyloid deposits accumulate at intervertebral discs, facet joints, and the ligamentum flavum in spine lesions and play an important role in the progression of spinal destruction and severe instability [2]. DSA was reported in approximately 20% of long-term dialysis patients [3,4]. With life expectancy increasing, it is inevitable that the number of long-term dialysis patients will also increase, which in turn will increase the number of DSA patients. Therefore, as the number of DSA patients continues to increase, the number requiring surgical treatment is also on the rise. In a report that compared DSA and non-DSA cases, operative time was significantly longer and blood loss was significantly greater in DSA cases [5]. Recently, lateral lumbar interbody fusion (LLIF) has become established as an alternative to conventional anterior and posterior fusion procedures for lumbar degenerative disease [6,7]. This procedure is a minimally invasive surgery that offers several potential advantages over traditional posterior approaches, including reduced blood loss and operative time [7]. Furthermore, LLIF with a percutaneous pedicle screw (PPS) has been reported as a minimally invasive technique for the treatment of degenerative diseases of the lumbar spine with good clinical results [8,9]. As far as we have been able to find out, there are no reports in English on the usefulness of this procedure for dialysis-related spondyloarthropathy; furthermore, the potential problems associated with its application in this indication remain undefined. In this study, we compared the clinical outcomes of LLIF with PPS in patients with dialysis-related spondyloarthropathy and lumbar degenerative disease, and discussed the challenges of this procedure in dialysis-related spondyloarthropathy.

## 2. Materials and Methods

### 2.1. Ethics Approval

Our Ethics Committee approved this study (14-02) and we followed the guidelines and regulations of the Ethics Review Committee for all methods. Because this is a retrospective study, the requirement of informed consent was waived.

### 2.2. Included Patients

We investigated the clinical outcomes of the LLIF (NuVasive Inc., San Diego, CA, USA) with PPS (ZimVie Inc., Westminster, CO, USA) procedure for dialysis-related spondyloarthropathy and lumbar degenerative disease. We retrospectively compared the clinical parameters of two groups: dialysis-related spondyloarthropathy (“Group D”) and lumbar degenerative disease (“Group L”) patients. From April 2014 to December 2021, 46 and 427 patients underwent surgery in Group D and Group L, respectively, including 19 and 117 patients who underwent LLIF with PPS. Of these cases, those with three or fewer vertebrae without direct decompression who could be followed up for at least one year after surgery were included in the study (Table 1).

### 2.3. Classification of Group D

**Figure 1:**
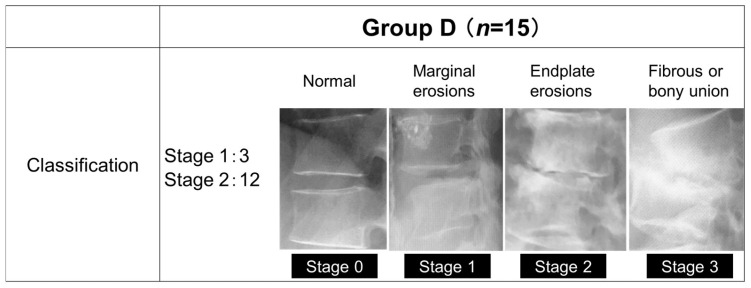
The progression classification of destructive spondyloarthropathy.

Group D consisted of 15 patients (10 males and 5 females) with a mean age of 70.4 years and a mean duration of hemodialysis of 10.8 years, and Group L consisted of 62 patients (31 males and 31 females) with a mean age of 71.0 years. The mean follow-up period was 4 years in group D and 3 years and 9 months in group L. The primary diseases that led to dialysis were diabetic nephropathy in 6 patients, chronic glomerulonephritis in 3 patients, nephrosclerosis in 3 patients, and unknown in 3 patients. The staging of group D was based on their progression classification of destructive spondyloarthropathy [10] (Figure 1), with Stage 0 being normal, Stage 1 being bone erosion of the anterior corner angle, Stage 2 being bone erosion of the vertebral body endplate and narrowing of the disc space, and Stage 3 being a loss of disc space and fusion of the vertebral body.

### 2.4. Variables

**Figure 2:**
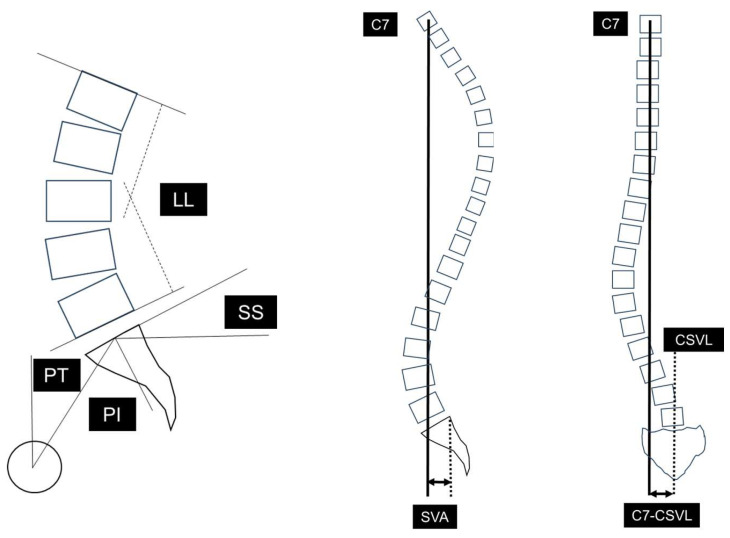
Radiographic spinopelvic parameters. PI: pelvic incidence, SS: sacral slope, PT: pelvic tilt, LL: lumbar lordosis, SVA: sagittal vertical axis offset, C7-CSVL: C7 plumb line-central sacrum vertical line.

We compared surgical invasiveness (operative time, blood loss), perioperative complications, clinical outcome, bone fusion rate (definition of bone fusion: continuity of cage and vertebral body endplate on computed tomography [CT] images 1 year after surgery, and bony bridging between vertebral bodies), and reoperations between the two groups. Clinical outcome was evaluated preoperatively, at the last follow-up, using the Japanese Orthopaedic Association (JOA) score for degenerative lumbar diseases (overall score: 29), which includes subjective symptoms and objective clinical signs [11]. The improvement ratio of the JOA score (%) was calculated according to the following formula: (postoperative (post.) JOA score − preoperative (pre.) JOA score)/(29 − pre. JOA score) × 100. Sagittal alignment (pre. and post.), including pelvic tilt (PT), sacral slope (SS), pelvic incidence (PI), angle of lumbar lordosis (LL), and sagittal vertical axis offset (SVA) were measured according to a report by Duval et al. [12]. Coronal imbalance (pre. and post.) included C7-CSVL (deviation of the C7 plumb line from the central sacral vertical line; this value was calculated by defining the convex side of CSVL using positive numerical values and the concave side using negative numerical values) [13,14] (Figure 2). PT corresponds to the angle between the line connecting the midpoint of the sacral plate to the HA and the vertical plane. SS was regarded as the angle between the superior endplate of S1 and the horizontal plane. PI was defined as the angle between the line perpendicular to the sacral plate and the line connecting the midpoint of the sacral plate to the hip axis (HA). LL was measured between the upper endplate of L1 and the lower endplate of L5. SVA was measured as the offset from the C7 vertebral plumb line and the superior posterior corner of the sacrum.

Dual-energy X-ray absorptiometry (DEXA) and serum-corrected calcium (Ca), phosphorus (P), and intact-parathyroid hormone (PTH) levels were measured in Group D to describe their bone metabolism status.

For statistical analysis, the Mann–Whitney U-test test was used, and *p* < 0.05 was considered a significant difference.

## 3. Results

The mean operative time per vertebral segment was 91.8 min (range: 46.7–183 min) for Group D and 107 min (range: 50.5–169 min) for Group L. The mean blood loss per vertebral segment was 63.4 mL (range: 10–227 mL) for Group D and 81.4 mL (range: 0–1185 mL) for Group L. There were no significant differences between the groups for these parameters. One patient in Group L required blood transfusion due to massive bleeding from a branch of their lumbar artery, but none of the other patients required transfusion. Perioperative complications in Group D included superficial infection in one patient (diabetic nephropathy, Stage 2) (6.7%) and endplate failure in three patients (diabetic nephropathy, Stage 1: one patient, diabetic nephropathy, Stage 2: one patient, chronic glomerulonephritis, Stage 2: one patient) (20.0%); in Group L, deep infection occurred one patient (1.6%) and massive bleeding in one patient (1.6%). Three patients (diabetic nephropathy, Stage 2: one; chronic glomerulonephritis, Stage 2: one; nephrosclerosis, Stage 2: one) (20.0%) in Group D and eleven patients (18.0%) in Group L experienced LLIF-specific transient neuropathy in the thigh (Table 2).

All the cases of approach-related complications such as hip flexor weakness or thigh pain/numbness were resolved spontaneously within 3 months post operation.

**Figure 3:**
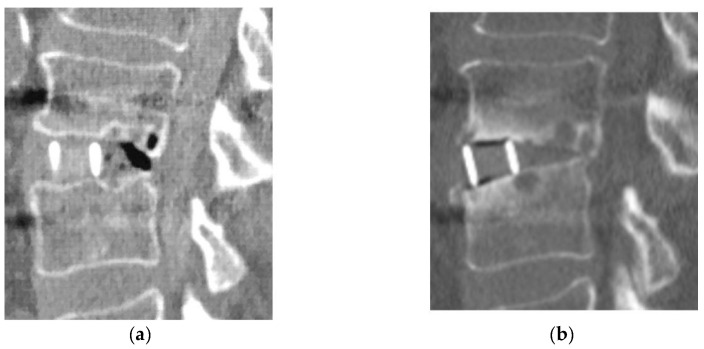
Reoperation case in Group D. (**a**): Immediate post-operative CT image; (**b**): 2-year postoperative CT image.

For clinical outcomes, the improvement ratio of the JOA score was 50.9% (−26.3–81.0%) in Group D and 57.9% (0–84.2%) in Group L, with no significant between-group difference. Bone fusion was observed in 11 of 15 patients (73.3%) in Group D and 56 of 62 patients (90.3%) in Group L. All four patients in Group D who did not achieve bone fusion had bone cysts around the cage and a loosening of the screws. One patient in Group D (diabetic nephropathy, Stage 2) (6.7%) had restenosis due to cage subsidence 2 years after LLIF with PPS and underwent additional decompression (Table 3) (Figure 3).

Table 4 shows the radiographic parameters. The PT (pre.) in Groups D and L was 26.5 (16–36) and 27.8 (12–49) degrees. The PT (post.) in Groups D and L was 25.8 (17–34) and 28.7 (4–48) degrees. The SS (pre.) in Groups D and L was 21.6 (16–34) and 22.5 (3–37) degrees. The SS (post.) in Groups D and L was 23.3 (12–30) and 23.4 (9–37) degrees. The PI (pre.) in Groups D and L was 45.9 (39–55) and 49.4 (39–66) degrees. The PI (post.) in Groups D and L was 46.3 (38–53) and 51.0 (38–68) degrees. The LL (pre.) in Groups D and L was 20.5 (9–37) and 22.6 (−13–45) degrees. The LL (post.) in Groups D and L was 19.6 (3–36) and 24.3 (−11–51) degrees. The mismatch between the PI and the LL (PI-LL) (pre.) in Group D was 25.4 (10–43) degrees. The mismatch between the PI and the LL (PI-LL) (pre.) in Group L was 26.8 (−2–60) degrees. The mismatch between the PI and the LL (PI-LL) (post.) in Group D was 26.6 (5–43) degrees. The mismatch between the PI and the LL (PI-LL) (post.) in Group L was 26.7 (3–59) degrees. The SVA (pre.) in Groups D and L was 64.7 (0–122) and 54.0 (−21–141) mm. The SVA (post.) in Groups D and L was 97.9 (0–222) and 58.2 (−16–195) mm. The C7-CSVL (pre.) in Groups D and L was 3.6 (−28–49) and 3.0 (−96–47) mm. The C7-CSVL (post.) in Groups D and L was 4.2 (−22–32) and 0.9 (−82–62) mm.

In Group D, the Young Adult Mean (YAM) DEXA values averaged 103% (71–137%) for the lumbar spine and 84% (55–104%) for the hip, with 4 of the 15 cases having a value below 70%. The mean serum Ca was 8.7 mg/dL (7.2–9.7 mg/dL) (normal range: 8.8–10.4 mg/dL), and there were no cases of hypocalcemia (i.e., <3.5 mg/dL). The mean serum P was 5.1 mg/dL (3.7–9.7 mg/dL) (normal range: 2.5–4.5 mg/dL), and hyperphosphatemia of 4.5 mg/dL was observed in 8 of 15 patients. Preoperative intact PTH averaged 147 pg/mL (25–551 pg/mL) (normal range: 10–65 pg/mL), and was within the normal range in 5 of 15 patients.

## 4. Discussion

Problems during the surgical treatment of dialysis spondylolisthesis include bleeding, infection, and bone fragility [15,16]. Chikawa et al. [5] reported that, in patients with lumbar spinal lesions, there were significant differences in the operative time and estimated blood loss between DSA and non-DSA groups. Chikuda et al. [15] found that the presence of destructive spondyloarthropathy was associated with the use of blood transfusions and longer anesthesia time, indicating that more complex surgical procedures were performed in this subgroup. In this study, of all 77 cases only 1 required blood transfusion, and it was a case from Group L. Both the operative time and blood loss for the LLIF with PPS procedure were comparable for Group D and Group L. The operative time and blood loss for Group D were significantly less than other previous studies [17,18,19]. Du et al. [20] compared the clinical results of OLIF and TLIF, with OLIF shortening the duration of surgery and reducing blood loss. Therefore, we consider this procedure useful in dialysis patients because of its minimally invasive technique.

Spinal surgery has historically had higher infection rates than other orthopedic surgeries. The infection rate of spinal surgeries reported in the literature ranges from 0.7 to 11.9% depending on the diagnosis and the complexity of the procedure [21,22]. Dialysis patients are at a high risk for infection because of their weak self-defense mechanisms and comorbidities [23]. In this study, deep infection was observed in one case in Group L and superficial infection in one case in Group D. Although there was no deep infection in Group D, the sample size was small (15 cases), and infection risk should be considered, with sufficient caution taken during the surgical procedure.

Although the improvement ratio of the JOA score of Group D was comparable with previous reports [17,18], it was worse than that in Group L. The reasons for this difference may be related to pseudarthrosis, endplate failure, and even bone fragility. In this study, endplate failure was observed in three patients in Group D and pseudoarthrosis in three patients. The bone fusion rates in previous studies investigating lumbar fusion surgery for patients with hemodialysis were 60–84% [16,17,18]. Similarly, the bone fusion rate for Group D in the present study was 73.3% for the 1-year follow-up period.

Teriparatide, a parathyroid hormone drug with osteogenic activity, may increase the bone remodeling [24] and bone healing [25] effects in hemodialysis patients with low intact PTH levels; it has also been reported that bone formation and bone turnover, leading to increased trabecular bone mass effects, may be achieved [26]. We think that teriparatide treatment may be an aid to bone fragility in hemodialysis spondylolisthesis. Furthermore, regarding the effects of teriparatide on Ca, P, and intact PTH, a study by Giamalis et al. [27] found that intact PTH levels were significantly increased, but that there was no significant difference in Ca and P levels after teriparatide treatment. This indicates that teriparatide had no effect on ectopic calcification. Therefore, the effect of teriparatide treatment on bone metabolism is considered to be small. In this study, 5 of the 15 patients had intact PTH levels within normal range, and 2 of these 5 patients had pseudoarthrosis. In patients with relatively low intact PTH levels, i.e., low bone turnover, it may be advisable to consider using the combination as pre- and postoperative adjunctive therapy, after careful evaluation of their intact PTH levels, their effect on bone metabolism, and blood pressure fluctuations, in view of bone fragility. In addition, Sato et al. [28] reported that BMD was increased significantly during romosozumab (a humanized antisclerostin antibody) treatment in the lumbar spine and the femoral neck, respectively, for 1 year in HD patients, and hypocalcemia occurred but without any intolerable events, while there was no apparent increase in CVD events during the 1 year of study. We think that romosozumab, as well as teriparatide, deserves to be used in combination as pre- and postoperative adjuvant therapy.

Although this procedure was not performed to treat sagittal alignment and coronal imbalance in adult spinal deformity, we examined pre- and post-surgery sagittal alignment and coronal imbalance.

Sagittal alignment showed no significant difference preoperatively and significant difference postoperatively for PT, PI, LL, and SVA. The reason for this difference may be related to dialysis changes during the follow-up period. Coronal imbalance showed no significant differences.

The reoperation rate for HD patients in the current study was 17.2%, which was significantly higher than that of non-HD patients [17]. In HD patients, Yamada T et al. reported that 8 of 29 patients (27.6%) needed revision surgery after lumbar fusion or decompression surgery [19]. In this study, 1 of 15 patients in Group D required reoperation during the follow-up period. Postoperative restenosis due to cage subsidence was observed in one patient. It was a reoperation case. One of the characteristics of LLIF is that indirect decompression can be obtained [29,30], but, on the other hand, reoperation due to insufficient indirect decompression caused by endplate failure has been noted as an issue with LLIF [31]. Indications for indirect decompression for hemodialysis spondylolisthesis should be carefully selected in light of amyloid deposition and the progression of postoperative destructive bone lesions; in some cases, additional direct decompression may be advisable.

We consider this procedure useful in dialysis patients because of its minimally invasive technique. Furthermore, although LLIF has the potential risk of several complications unique to the procedure, no major complications such as major vascular injury, ureteral injury, or bowel injury occurred in this study. We believe that LLIF with PPS for dialysis-related spondyloarthropathy can also be performed minimally invasively and safely; on the other hand, endplate failure due to bone fragility and a reduced bone fusion rate were observed in dialysis spondylolisthesis cases. Postoperative management basically involves wearing a rigid orthosis for approximately 3 months, but further study is needed to determine the best duration of its wear and the type of orthosis required. We believe that this procedure is an effective treatment option for dialysis spondylolisthesis, but that the indication for indirect decompression should be carefully considered, and pre- and postoperative adjuvant therapies should be devised.

This study has several limitations. First, it was confined to a single-center design, which may affect the generalizability of our findings to other institutions with different surgical practices and patient demographics. Second, this study was retrospective, with a small sample size, which allows for the potential for selection bias, affecting our control over the data collection and interpretation of causal relationships. Moreover, there are few studies with larger sample sizes that investigate the clinical outcomes of spinal surgery in HD patients. Third, this single-arm study had no control group and patient-reported outcomes. Studies with a larger sample size, long-term follow-up, and clinical evaluation are needed. Fourth, regarding sample size analysis, this study is retrospective, not prospective, and thus this was not conducted for this study. In future efforts, we would need to increase the sample size and conduct multi-centered studies, which could enhance the generalizability of the results. Based on this study, endplate failure and the bone fusion rate due to the use of teriparatide or romosozumab should also be studied in the future.

## 5. Conclusions

We consider LLIF with PPS for dialysis-related spondyloarthropathy to be an effective treatment option because its surgical invasiveness and clinical outcomes were comparable to those for cases of lumbar degenerative disease. However, as endplate failure due to bone fragility and a reduced bone fusion rate were observed in dialysis spondylolisthesis cases, we advise a careful selection of indications for indirect decompression as well as the development and application of suitable pre- and postoperative adjuvant therapies.

## Figures and Tables

**Table 1:** Clinical characteristics of subjects in the dialysis-related spondylolisthesis (Group D) and lumbar degenerative disease (Group L) groups.

	Group D (*n* = 15)	Group L (*n* = 62)
Sex	Male: 10, Female: 5	Male: 31, Female: 31
Age (y)	70.4 (60–78)	71.0 (66–83)
Follow-up duration (mo)	48 (19–96)	44 (12–98)
Duration of hemodialysis (y)	10.8 (2–29)	

**Table 2:** Surgical invasiveness and perioperative complications in Group D and Group L.

	Group D (*n* = 15)	Group L (*n* = 62)	*p* Value
Operative time (min)		104 (50.5–169)	0.115
	91.8 (46.7–183)		
Blood loss (mL)	63.4 (10–227)	81.1 (0–1185)	0.877
Perioperative complications	Superficial infection (6.7%)	Deep infection (1.6%)	
	Endplate failure (20.0%)	Massive bleeding (1.6%)	
	Transient neuropathy in the thigh (20.0%)	Transient neuropathy in the thigh (18.0%)	

**Table 3:** Clinical outcomes and bone fusion rates in Group D and Group L.

	Group D (*n* = 15)	Group L (*n* = 62)	*p* Value
Clinical outcome (improvement ratio of the JOA score (%))		57.9 (0–84.2)	0.944
	50.9 (−26.3–81)		
Reoperation	1	1	
Bone fusion rate (%)	73.3	90.3	0.079

**Table 4:** Sagittal alignment and coronal imbalance in Group D and Group L. Preoperative and postoperative spinopelvic parameters of the two groups.

	Group D (*n* = 15)	Group L (*n* = 62)	*p* Value	Group D (*n* = 15)	Group L (*n* = 62)	*p* Value
	Pre.	Pre.		Post.	Post.	
PT (degree)	26.5 (16–36)	27.8 (12–49)	0.119	25.8 (17–34)	28.7 (4–48)	0.035 *
SS (degree)	21.6 (16–34)	22.5 (3–37)	0.114	23.3 (12–30)	23.4 (9–37)	0.210
PI (degree)	45.9 (39–55)	49.4 (39–66)	0.059	46.3 (38–53)	51.0 (38–68)	0.022 *
LL (degree)	20.5 (9–37)	22.6 (−13–45)	0.119	19.6 (3–36)	24.3 (−11–51)	0.040 *
PI-LL (degree)	25.4 (10–43)	26.8 (−2–60)	0.882	26.6 (5–43)	26.7 (3–59)	0.879
SVA (mm)	64.7 (0–122)	54.0 (−21–141)	0.416	97.9 (0–222)	58.2 (−16–195)	0.049 *
C7-CSVL (mm)	3.6 (−28–49)	3.0 (−96–47)	0.189	4.2 (−22–32)	0.9 (−82–62)	0.215

PT: pelvic tilt, SS: sacral slope, PI: pelvic incidence, LL: lumbar lordosis, SVA: sagittal vertical axis offset, C7-CSVL: C7 plumb line-central sacrum vertical line. * *p* < 0.05 statistically significant.

## Data Availability

The data presented in this study are available on request from the corresponding author. The data are not publicly available due to respect for patient privacy.
